# The association of mast cells and serotonin in children with chronic abdominal pain of unknown etiology

**DOI:** 10.1186/1756-0500-3-265

**Published:** 2010-10-21

**Authors:** Tara J Taylor, Nader N Youssef, Ravi Shankar, David E Kleiner, Wendy A Henderson

**Affiliations:** 1 National Institute of Nursing Research, National Institutes of Health, Bethesda, MD, USA; 2 Center for Pediatric Functional Gastrointestinal and Motility Disorders, Goryeb Children's Hospital at Atlantic Health, University of Medicine & Dentistry of New Jersey, Morristown, NJ, USA; 3 National Cancer Institute, National Institutes of Health, Bethesda, MD, USA

## Abstract

**Background:**

Abdominal pain of unknown origin affects up to 20% of school-aged children. Evaluation of children is symptom-based without clear guidelines to investigate molecular mechanisms of abdominal pain. Aberrant molecular mechanisms may increase intestinal permeability leading to interactions between the immune and nervous systems, subclinical inflammation, and visceral pain. This study evaluated the association between interleukin-6 (IL-6), mast cell infiltrates, and serotonin (5-HT) levels in gastrointestinal (GI) biopsies, with perceived abdominal pain in a pediatric cohort.

**Methods:**

Clinical data and biopsy samples from pediatric patients (*n *= 48) with chronic abdominal pain, with and without inflammation were included. Formalin-fixed paraffin-embedded GI biopsies were sectioned and immunohistochemistry performed for IL-6 and 5-HT; mast cells were identified with toluidine blue stain. Histological findings were compared to self-reported abdominal pain between groups.

**Results:**

There was significantly greater IL-6 immunoreactivity in biopsies with confirmed histologic inflammation (*p *= 0.004). There was a greater number of mast cells per HPF in non-inflammatory biopsies (3.5 ± 2.9) compared to the inflammatory biopsies (2.6 ± 1.8) *p *= 0.049. The non-inflammatory biopsy group was significantly less likely to respond to standard treatment as evidenced by higher pain reports (*p *= .018). Mast cells (*p *= .022) and 5-HT (*p *= .02) were significantly related to abdominal pain scores.

**Conclusions:**

A potential association between self-reported abdominal pain, number of mast cells, and 5-HT levels, which may contribute to perceived GI pain in pediatric patients may exist.

## Background

Unspecified chronic abdominal pain in children that has no identified biologic marker or known organic cause and has occurred for greater than two months is defined by Rome III criteria as chronic abdominal pain [1]. These criteria assume that no metabolic or structural causes can be related to the continued symptoms of chronic abdominal pain for an eight week period over the previous year. Current Rome III criteria divide pediatric abdominal pain diagnosis into several subsets which include functional abdominal pain syndrome and irritable bowel syndrome (IBS) [2]. Approximately 15-20% of children [3] and adults living in the United States suffer from chronic abdominal pain [4,5]. Children with chronic abdominal pain experience lessened quality of life in comparison to their healthy peers which leads to numerous absences from school for medical care [6,7]. Chronic abdominal pain in pediatric patients is a significant burden on the health system which warrants further investigation on the pathogenesis of disease to identify novel targets for intervention.

Recognition of the role of inflammation and its interaction with the neuro-immune system of the gastrointestinal tract is an emerging area of interest in patients with chronic abdominal pain [8]. The mechanisms of chronic abdominal pain of unknown origin may be related to interactions between the immune and nervous system in the gut thereby leading to visceral hypersensitivity of the intestinal mucosa [8]. Mast cells have been shown to interact with colonic nerve endings by the secretion of tryptase, histamine, and possibly serotonin (5-hydroxytryptamine, 5-HT) in humans [9]. There is evidence of closer proximity of mast cells to nerve fibers in the colon of IBS patients leading to augmented visceral sensitivity [10]. A possible relationship between the number of mucosal mast cells and rectal sensitivity has also been demonstrated in humans [11]. There is evidence of a significant increase in mast cell numbers in patients with IBS [12]. Along with increased mast cell counts there is support that mast cell numbers directly correlate with abdominal pain in IBS patients [13]. Recently, a study conducted by Mahjoub, et al. found increased mast cell density in pediatric patients with recurrent abdominal pain [14]. They propose that mast cell density measurements be incorporated with routine GI biopsies due to a significant correlation between increase GI complaints and mast cell count. Inflammation and increased permeability of GI tract mucosa may also induce pain in pediatric patients with functional abdominal pain [15].

Chronic abdominal pain of unknown origin may be due to low grade inflammatory changes in the GI tract [16,17]. Low level inflammation may result in increased permeability across the mucosal barrier; which in turn permits the entrance of antigens into the intestinal wall [16]. Other potential mechanisms include altered function at the level of neurotransmitter receptors of 5-HT, increased visceral hypersensitivity, and impaired colonic mucosal permeability [18]. Alleviation of symptoms with medication that target 5-HT receptors suggest that 5-HT is involved in gut motility regulation [19].

Increased permeability and its role in pediatric abdominal pain has recently been the focus of work by Shulman (2008). They explored the relationship of subclinical inflammation and its relationship to chronic pain of the GI tract. In a well-designed prospective controlled study investigating the difference in GI permeability and fecal calprotectin (marker for intestinal inflammation) concentration in children with abdominal pain versus control [15]. Proximal GI permeability, colonic permeability, and fecal calprotectin were found to be significantly greater in the abdominal pain group compared to the control group. Fecal calprotectin concentrations correlated with pain interference with activities. However, there was no correlation between GI permeability and pain-related symptoms. This study highlighted that more research is needed to examine the interactions at the molecular level between mast cells and 5-HT.

These converging lines of evidence suggest that mast cells and 5-HT contribute to increased colonic permeability and thereby lead to chronic abdominal pain. We assessed this hypothesis at the molecular level by exploring the relationship between mast cell and 5-HT levels and perceived abdominal pain in a pediatric cohort with and without the diagnosis of an inflammatory GI disorder.

## Methods

### Patients

This retrospective study sample included pediatric patients who had undergone an initial outpatient GI and endoscopic evaluation with biopsies as part of routine evaluation for persistent abdominal pain and other associated symptoms (Table 1). After diagnostic evaluation, patients were classified as having abdominal pain that was related to gross inflammation such as Crohn's disease or ulcerative colitis and whose pain subsequently resolved on anti-inflammatory therapies versus those with no evidence of both gross and histologic inflammation who continued to have abdominal pain. These patients were considered to have functional abdominal pain consistent with Rome III criteria. Formalin-fixed paraffin-embedded biopsies (*n *= 48) from the esophagus (4), antrum (1), stomach/gastric body (7), duodenum (3), and colon/cecum (33) were available for pathologic evaluation. A selection of samples from the prior 3 years was purposely selected by a blinded pathologist across phenotype, gender and age. The biopsies and data collected were de-identified and received through a material transfer agreement approved by the Institutional Review Board (IRB) of Goryeb Children's Hospital at Atlantic Health, Morristown, New Jersey with the National Institutes of Health (NIH).

**Table 1:** Demographic and clinical indicators of sample.

Variable		Overall (*n *= 48)	Group All subjects were pain positive		Statistic
				
			Non-Inflammatory (*n *= 26)	Inflammatory (*n *= 22)	
		***n *(%)**	***n *(%)**	***n *(%)**	***p *value** **(χ**^**2 **^**/t-test)**

**Sex**					

	Male	22 (45.8)	11	11	**0.59**

	Female	26 (54.2)	15	11	

**Race**					

	Caucasian	43 (89.6)	23	20	**0.58**

	Asian	2 (4.2)	1	1	

	African American	1 (2.1)	1	0	

	Mixed	1 (2.1)	0	1	

	Hispanic	1 (2.1)	1	0	

**Age (M ± SD)** **Range (yrs)**		11.9 ± 2.9 (5-17)	11.9 ± 2.4 (8-16)	12 ± 2.9 (5-17	**0.21**

**BMI (M ± SD)** **Range**		18.9 ± 4.3 (13-38.8)	19.5 ± 5.2 (13.8-38.8)	18.2 ± 4.3 (13-24.5)	**0.431**

**Lactase Deficiency**		24 (48)	16	8	**0.387**

### Clinical data collection

All abdominal pain reports (categorized as no pain, mild, moderate, or severe) were recorded before and after 6 months of endoscopic examination and medical treatment as a part of routine clinical care. All patients reported abdominal pain for more than 6 months prior to endoscopy. Pre-existing data were extracted and re-coded into a secure database without personal identifiers. Clinical data included sex, race, age, body mass index (BMI), and lactase deficiency. Data on race were collected by electronic medical record review and categorized as Caucasian, Asian, African American, Hispanic, and mixed race. BMI was calculated from the initial outpatient visit information with the formula age (years) by weight (kilograms)/height (meters) squared. Medical record data of confirmed lactase deficiency was collected and categorized as positive or negative based on a duodenal biopsy disaccharidase assay (Women's and Children's Hospital of Buffalo, Buffalo, NY).

### Pathological examination

Biopsies were stained with hematoxylin and eosin, toluidine blue and immunohistochemical markers at the National Cancer Institute, Science Applications International Corporation (Frederick, MD). Microscopic histologic review of stains was performed by either a pathologist or trained technicians who were blinded to clinical information. Biopsies were categorized as either inflamed (with evidence of chronic mucosal changes, villous/mucosal atrophy, ulceration, cryptitis, crypt abscesses, or crypt destruction) or not inflamed.

Mast cells identification was performed after the biopsies were sectioned (5-6 microns), de-paraffinized and rinsed with 60% ethanol. Slides were then stained in toluidine blue for 2 minutes, rinsed, and dehydrated in acetone (2 times for 2 minutes). Slides were cleared in xylene and mounted. Mast cell number was recorded as the number of cells per 40× high power field (HPF) then averaged 10 fields containing the maximum number of mast cells.

For 5-HT immunohistochemistry, colonic GI tract biopsies were sectioned (7 microns), de-paraffinized, and incubated with 2% normal horse serum (Vector Laboratories Inc. Burlingame, CA) for 20 minutes followed by incubation with anti-5-HT antibody diluted 1:40 (Vision Biosystems, Newcastle, UK) for 30 minutes at room temperature. Slides were rinsed and biotinylated horse anti-mouse IgG antibody (Vector Laboratories Inc., Burlingame, CA) was applied for 30 minute incubation. Immunoreactivity of 5-HT was identified by positive stained entrochromaffin (EC) cells. The slides were scored as: minimal (1-2 EC), mild (3-5 EC), moderate (6-10 EC), or marked (more than 16 EC) by a pathologist.

For IL-6 immunohistochemistry, biopsies were sectioned (7 microns), de-paraffinized and blocked with 2% normal goat serum (Vector Laboratories Inc. Burlingame, CA) for 20 minutes followed by incubation with anti-IL-6 antibody diluted 1:400 (Vision Biosystems, Newcastle, UK) at room temperature for 30 minutes. Slides were then incubated for 30 minutes with biotinylated goat anit-rabbit IgG (Vector Laboratories Inc., Burlingame, CA). The level of IL-6 immunoreactivity was scored as: minimal, mild, moderate, or marked by a pathologist.

### Statistical methods

Data were collected, coded and doubly entered into SPSS version 15.0. Statistical analysis included: means, frequencies, standard deviation, independent *t *test and Chi square analysis.

### Ethical considerations

This study was approved by the NIH Office of Human Subject Research (protocol #3906) and the Institutional Review Board of Goryeb Children's Hospital- Atlantic Health (IRB #R07-09-009).

## Results

The overall sample included pediatric patients (*n *= 48), 54% female, with a mean age of 11.9 ± 2.9 yrs (range 5-17 yrs). After histological review, 22 pediatric GI biopsies were categorized as inflammatory and 26 pediatric GI biopsies as non-inflammatory which coincided with the original clinical diagnosis of disease (inflammatory bowel disease, gastritis, IBS, or functional abdominal pain). There was no significant difference between the phenotype (non-inflammatory and inflammatory GI mucosa) with regard to sex, race, age, BMI or lactase deficiency (Table 1).

**Figure 1:**
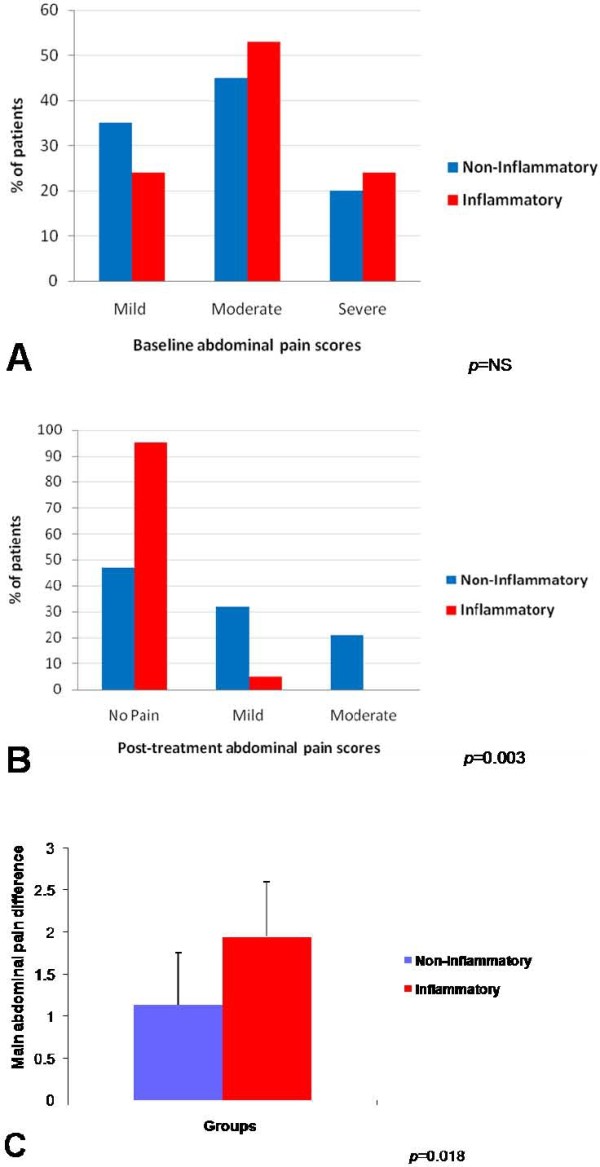
**Self-reported abdominal pain scores**. Self-reported abdominal pain scores were dependent on the phenotype of non-inflammatory or inflammatory. **A**. At baseline there is no significant difference of self-reported abdominal pain between groups. **B**. At follow-up patients with inflamed biopsies reported lower pain scores. **C**. Patients with non-inflamed biopsies had less change at baseline compared to follow-up self reported pain scores.

Although there was no significant difference between the patients abdominal pain reports at baseline (χ^2 ^(2) = 0.58; *p *= 0.75) (Figure 1A), the response to standard care was significantly related to the histology of the patient's biopsy as evidenced by patients with non-inflamed biopsies reporting significantly higher pain reports than patients with inflamed biopsies following endoscopy/medical treatment (*χ*^2 ^(2) = 11.67; *p *= 0.003) (Figure 1B). The difference in the abdominal pain reports (pre and post endoscopy/medical treatment) differed significantly between the two phenotypic groups (χ^2 ^(3) = 10.12; *p *= 0.018) (Figure 1C).

At baseline self-reported pain scores of patients with non-inflamed biopsies were 35% mild, 45% moderate, and 20% severe, compared to patients with inflamed biopsies 23.5% mild, 53% moderate, and 23.5% severe (Figure 1A). Post-treatment self-reported pain scores of patients with non-inflamed biopsies were 47% no pain, 32% mild pain, 21% moderate pain; patients with inflamed biopsies 95% reported no pain, 5% reported mild pain (Figure 1B). The difference between pre and post treatment self-reported pain scores for patients with non-inflamed biopsies were 12.5% reported no change in pain, 62.5% reported a mild change in pain, and 25% reported moderate change in pain. The difference in pre and post treatment self-reported pain scores of patients with inflamed biopsies were 23.5% mild change in pain, 58.8% reported moderate change in pain, 17.6% reported in a complete change in pain (Figure 1C).

**Figure 2:**
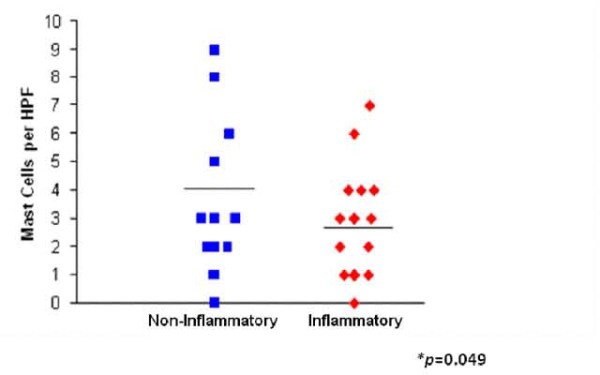
**Mast cells per HPF**. Increased number of mast cells in non-inflammatory biopsies compared to inflammatory biopsies.

**Figure 3:**
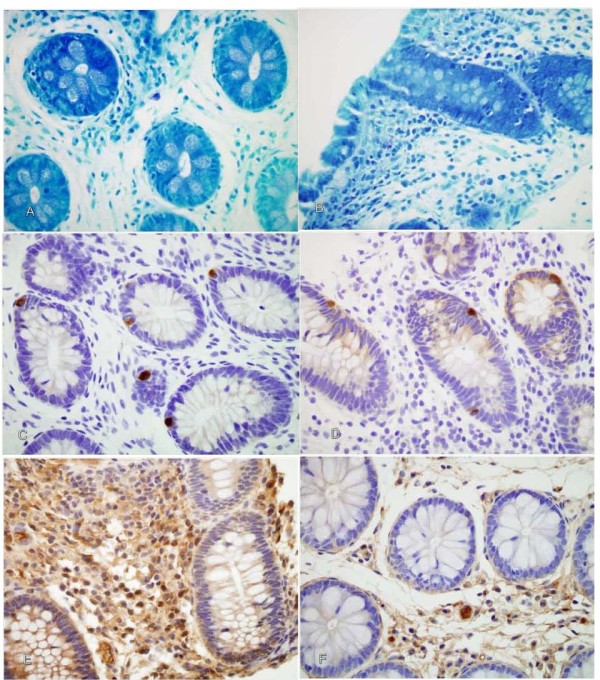
**Immunohistochemistry of mast cells, 5-HT, and IL-6 (magnification 60×)**. **A-F**. FFPE GI mucosa block sectioned and immunostained for mast cells, 5-HT, and IL-6. **A-B**. Increased mast cell counts per HPF in non-inflammatory biopsies compared to inflammatory biopsies. **C-D**. No significant difference between phenotypes in 5-HT immunoreactivity. **E-F**. Increased IL-6 immunoreactivity of inflammatory biopsies compared to non-inflammatory biopsies.

Evaluation of toluidine blue stained sections showed that there was a greater number of mast cells per HPF in non-inflammatory biopsies (3.5 ± 2.9) compared to the inflammatory biopsies (2.6 ± 1.8) *p *= 0.049 (Figure 2). The number of mast cells (*p *= 0.022) and immunoreactivity of 5-HT (*p *= 0.02) per HPF were significantly related to self-reported abdominal pain scores. Patients with non-inflamed biopsies that had increased levels of mast cells (Figure 3A and 3B) and 5-HT reported higher post-treatment abdominal pain scores compared to patients with inflamed biopsies (Figure 1B). Patients with non-inflamed biopsies that failed to respond to treatment (i.e., little change from baseline to post-treatment pain scores) had increased mast cells and 5-HT levels (mast cells *r *= -0.42 and 5-HT *r *= -0.52). Patients with inflamed biopsies that had increased levels of mast cells and 5-HT reported higher change in pain scores (*r *= 0.55). There was no difference in the amount of staining for 5-HT between inflamed and non-inflamed biopsies (*χ*^2 ^(3) = 4.08; *p *= 0.25) (Figure 3C and 3D). The inflammatory biopsies had increased levels of IL-6 immunoreactivity compared to the biopsies without inflammation (χ^2 ^(2) = 10.9; *p *= 0.004) (Figure 3E and 3F).

## Discussion

In this study, we illustrate for the first time in the pediatric population with chronic abdominal pain of unknown origin that increased self-reported abdominal pain is correlated with higher levels of mast cells and 5-HT. All patients, non-inflammatory and inflammatory, presented with abdominal pain prior to endoscopic evaluation. Patients with inflammation responded better to standard care as evidenced by lower self-reported abdominal pain scores post endoscopic evaluation. Patients with decreased pain after standard treatment had lower mast cell and 5-HT levels. The non-inflammatory cohort who were unresponsive to standard medical care presented with higher pain, had increased mast cells, and 5-HT levels compared to the inflammatory cohort. This early work may begin to explain some of the elusive etiology related to chronic abdominal pain of unknown origin in children.

**Figure 4:**
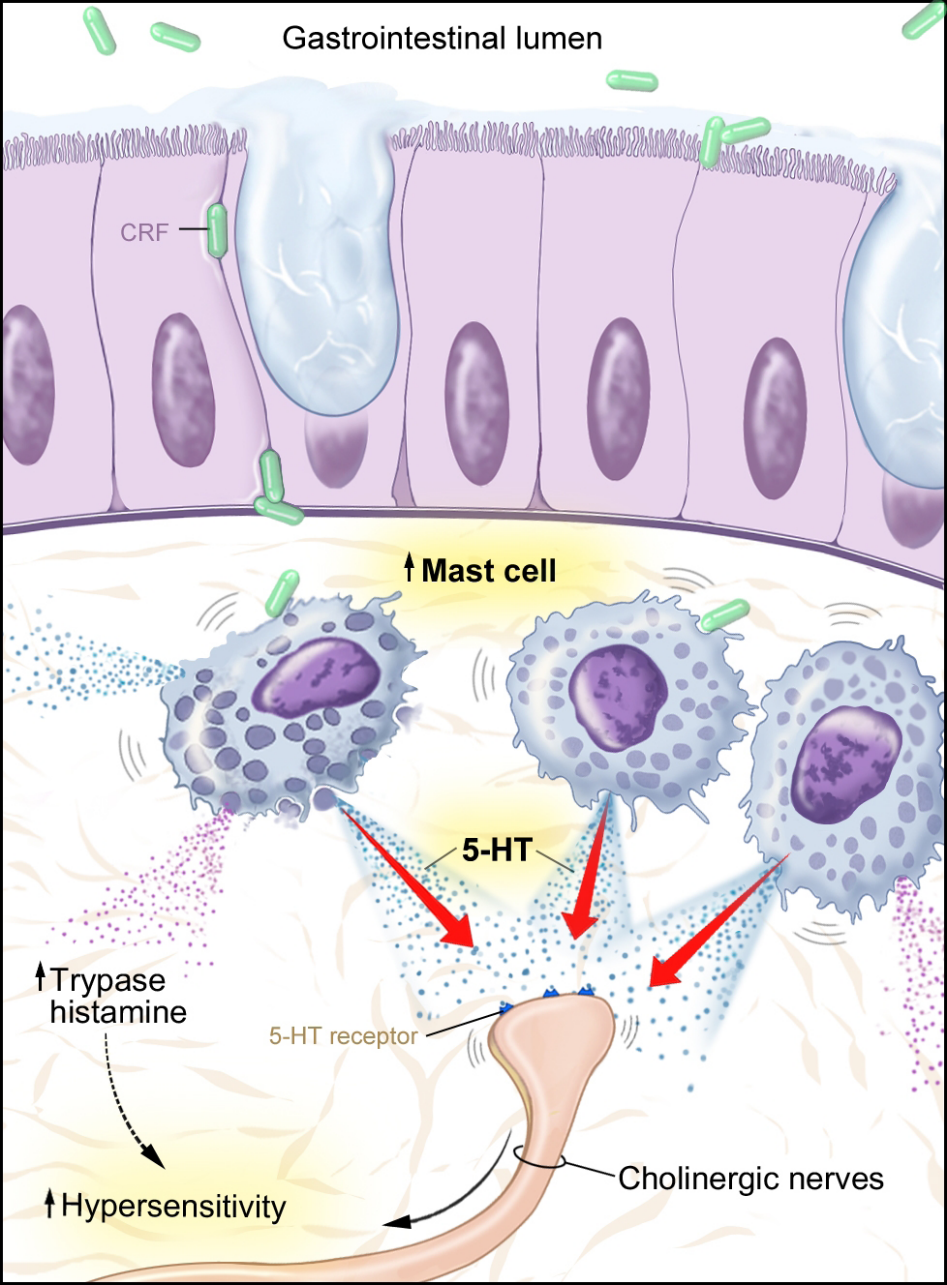
**Visceral activation of mast cells**. Visual depiction of 5-HT acting as a potential mediator between mast cells and cholinergic nerves yielding visceral hypersensitivity.

This research tested the hypothesis that chronic abdominal pain of unknown origin has an inflammatory basis due to visceral activation by mast cells in the gut (Figure 4). Mast cells not only degranulate and release pro-inflammatory substances but also may be in closer proximity to the cholinergic nerves thereby altering GI motility and hypersensitivity (i.e., increased abdominal pain) [10]. Mast cells may release other mediators besides histamine and typtase that affect visceral hypersensitivity. Our data suggests that 5-HT is an alternative mast cell mediator which could interact with peripheral nerves leading to increased sensitivity in the gut and chronic abdominal pain. Our findings of increased mast cell and 5-HT immunoreactivity propose that these two components of the immune system are related and contribute to chronic abdominal pain of unknown origin in children.

Current proposed mechanisms of IBS give additional information which may be related to the mechanism behind chronic abdominal pain of unknown origin. For example, in post infectious IBS there may be a translocation of microbiota due to increased mucosa permeability allowing for the interaction between inflammatory mediators and the enteric nervous system thereby stimulating smooth muscle motility [20]. Patients with chronic abdominal pain may be affected by a similar mechanism at the molecular level of the GI mucosa. Increased permeability across the mucosal barrier may occur, in turn leading to hypersensitivity and augmented pain.

### Limitations

There are some limitations to this study including the retrospective nature of data collection using medical record review. Additionally, the limited sample size makes the findings not necessarily generalizable to the greater population of patients with chronic abdominal pain of unknown origin. Furthermore, because of the retrospective nature only associations are presented here and care should be taken in the interpretation of these findings as they do not imply direct causality. A future study could include both direct objective and subjective measures of abdominal pain as well as real-time measures of 5-HT, IL-6, and mast cell activation.

## Conclusions

Recent advances on the role of 5-HT in the enteric nervous systems and its relationship to intestinal visceral hyperalgesia have contributed significantly to the understanding of abdominal pain and IBS in adults. This increased knowledge has helped shift the paradigm that these disorders are exclusively behavioral in nature and that pathophysiologic disturbances at the cellular level exist. To date, the consideration of inflammation and intestinal pain has been traditionally reserved for conditions such as Crohn's disease and ulcerative colitis. The preliminary findings presented in this manuscript indicate that similar relationships may exist in the pediatric population with chronic abdominal pain of unknown origin. Future research is needed in order to uncover the associated causes of abdominal pain without inflammatory pathology in pediatric patients. Novel medical treatments can arise by unveiling the role of mast cells and 5-HT in the pathophysiology of chronic abdominal pain of unknown origin in children.

## Abbreviations

GI: gastrointestinal; 5-HT: 5-hydroxytryptamine (serotonin); IBS: irritable bowel syndrome.

## Competing interests

The authors declare that they have no competing interests.

## Authors' contributions

TT, NY, RS, DK, WH: all contributed to both the manuscript development and analysis. All authors read and approved the final draft.

## References

[B1] Drossman D Corazziari E Delvaux M Spiller RC Talley NJ Thompson WG Whitehead WE Rome III; The Functional Gastrointestinal Disorders 2006 Third Degnon Associates, Inc.

[B2] Hahn B Saunders W Maier W Differences between individuals with self-reported irritable bowel syndrome (IBS) and IBS-like symptoms Dig Dis Sci 1997 42 12 2585 2590 10.1023/A:1018889318063 9440642

[B3] Petersen S Brulin C Bergström E Recurrent pain symptoms in young schoolchildren are often multiple Pain 2006 121 1-2 145 150 10.1016/j.pain.2005.12.017 16473464

[B4] Creed F Ratcliffe J Fernandez L Tomenson B Palmer S Rigby C Guthrie E Read N Thompson D Health-Related Quality of Life and Health Care Costs in Severe, Refractory Irritable Bowel Syndrome Ann Intern Med 2001 134 9_Part_2 860 868 11346322 10.7326/0003-4819-134-9_part_2-200105011-00010

[B5] Russo MW Gaynes BN Drossman DA A National Survey of Practice Patterns of Gastroenterologists With Comparison to the Past Two Decades Journal of Clinical Gastroenterology 1999 29 4 339 343 10.1097/00004836-199912000-00009 10599638

[B6] Youssef NN Murphy TG Langseder AL Rosh JR Quality of life for children with functional abdominal pain: a comparison study of patients' and parents' perceptions Pediatrics 2006 117 1 54 59 10.1542/peds.2005-0114 16396860

[B7] Whitehead W Burnett C Cook E Impact of IBS on quality of life Dig Dis Sci 1996 41 2248 2253 10.1007/BF02071408 8943980

[B8] De Giorgio R Barbara G Is irritable bowel syndrome an inflammatory disorder? Curr Gastroenterol Rep 2008 10 4 385 390 10.1007/s11894-008-0073-0 18627650

[B9] Barbara G Stanghellini V De Giorgio R Cremon C Cottrell GS Santini D Activated mast cells in proximity to colonic nerves correlate with abdominal pain in irritable bowel syndrome Gastroenterology 2004 126 3 693 702 10.1053/j.gastro.2003.11.055 14988823

[B10] Barbara G Wang B Stanghellini V de Giorgio R Cremon C Di Nardo G Mast cell-dependent excitation of visceral-nociceptive sensory neurons in irritable bowel syndrome Gastroenterology 2007 132 1 26 37 10.1053/j.gastro.2006.11.039 17241857

[B11] Park JH Rhee PL Kim HS Lee JH Kim YH Kim JJ Mucosal mast cell counts correlate with visceral hypersensitivity in patients with diarrhea predominant irritable bowel syndrome J Gastroenterol Hepatol 2006 21 1 Pt 1 71 78 10.1111/j.1440-1746.2005.04143.x 16706815

[B12] Piche T Saint-Paul MC Dainese R Marine-Barjoan E Iannelli A Montoya ML Peyron JF Czerucka D Cherikh F Filippi J Mast cells and cellularity of the colonic mucosa correlated with fatigue and depression in irritable bowel syndrome Gut 2008 57 4 468 473 10.1136/gut.2007.127068 18194987

[B13] Akbar A Yiangou Y Facer P Walters JR Anand P Ghosh S Increased capsaicin receptor TRPV1-expressing sensory fibres in irritable bowel syndrome and their correlation with abdominal pain Gut 2008 57 7 923 929 10.1136/gut.2007.138982 18252749 PMC2564830

[B14] Mahjoub FE Farahmand F Pourpak Z Asefi H Amini Z Mast cell gastritis: children complaining of chronic abdominal pain with histologically normal gastric mucosal biopsies except for increase in mast cells, proposing a new entity Diagn Pathol 2009 4 34 10.1186/1746-1596-4-34 19799799 PMC2761861

[B15] Shulman RJ Eakin MN Czyzewski DI Jarrett M Ou CN Increased gastrointestinal permeability and gut inflammation in children with functional abdominal pain and irritable bowel syndrome J Pediatr 2008 153 5 646 650 10.1016/j.jpeds.2008.04.062 18538790 PMC2614282

[B16] Barbara G Mucosal barrier defects in irritable bowel syndrome. Who left the door open? Am J Gastroenterol 2006 101 6 1295 1298 10.1111/j.1572-0241.2006.00667.x 16771952

[B17] Spiller R Garsed K Postinfectious irritable bowel syndrome Gastroenterology 2009 136 6 1979 1988 10.1053/j.gastro.2009.02.074 19457422

[B18] Crowell MD Role of serotonin in the pathophysiology of the irritable bowel syndrome Br J Pharmacol 2004 141 8 1285 1293 10.1038/sj.bjp.0705762 15100164 PMC1574906

[B19] Costedio MM Hyman N Mawe GM Serotonin and its role in colonic function and in gastrointestinal disorders Dis Colon Rectum 2007 50 3 376 388 10.1007/s10350-006-0763-3 17195902

[B20] Barbara G Cremon C Pallotti F De Giorgio R Stanghellini V Corinaldesi R Postinfectious irritable bowel syndrome J Pediatr Gastroenterol Nutr 2009 48 Suppl 2 S95 97 10.1097/MPG.0b013e3181a15e2e 19300138

